# Treatment regimens, patient reported outcomes and health-related quality of life in children with moderate and severe hemophilia A in China: using real-world data

**DOI:** 10.1186/s13023-023-02835-x

**Published:** 2023-08-04

**Authors:** Luying Zhang, Peng Zhang, Wen Chen

**Affiliations:** 1 School of Public Health, Fudan University, Shanghai, 200032 China; 2 School of Humanities, Shanghai Institute of Technology, Shanghai, 201418 China

**Keywords:** Hemophilia, Treatment regimen, Prophylaxis, Outcome, Health-related quality of life, Children

## Abstract

**Background:**

Prophylaxis therapy for children with moderate and severe hemophilia A (HA) is the optimal treatment regimen. The real-world treatment regimens, patient-reported outcomes, and health-related quality of life (HRQoL) in children with moderate and severe HA in China are less known.

**Objective:**

This study aimed to describe real-world treatment regimens and evaluate the association of treatment regimens with comprehensive patient-reported outcomes including bleeds, chronic pain, target joints, disability, and HRQoL in children under 18 years old with HA in China.

**Methods:**

Real-world data of a nationwide online cross-sectional survey in 2021 and patients’ coagulation factor utilization data from self-management records from 2020 to 2021 were merged. 373 eligible children were included and categorized by treatment regimens according to the Chinese guideline: on-demand, short-term prophylaxis, and long-term prophylaxis treatment.

**Results:**

Currently, in China, 4.8% of children with HA are receiving full-dose long-term prophylaxis treatment. Prophylaxis treatment was a significant positive predictor of better patient-reported outcomes and HRQoL. For children with prophylaxis treatment, there were significantly fewer annual bleeds (p < 0.001), lower frequency of chronic pain(p < 0.001), and higher health utility scores(p < 0.01) and EQ-VAS scores(p < 0.05) than children with on-demand treatment.

**Conclusion:**

Accessible long-term prophylaxis treatment should be promoted for children with moderate and severe HA in China and regular monitoring of their outcomes and HRQoL should be carried out.

## Background

Hemophilia is a rare, inherited bleeding disorder caused by a deficiency of coagulation factor VIII (FVIII) (in hemophilia A) or factor IX (in hemophilia B) [1]. Hemophilia A (HA) accounts for approximately 80–85% of hemophilia cases [2]. Hemophilia is associated with the symptoms of spontaneous bleeding at an early age, which can lead to long-term inflammation, joint destruction with a limited range of motion or even life-long disability and impairment [3]. Therefore, children with hemophilia must acquire adequate treatment to avoid bleeding complications and joint damage and enable them to reach adulthood as healthy as possible [1].

Continuous prophylaxis treatment is broadly recommended as the optimal regimen and the standard care for children with moderate and severe HA [1, 4]. Prophylaxis therapy for children has been widely adopted in Western countries including Germany, Canada, and the United States [5–7]. It is reported in Canada 77% of hemophilia children received prophylaxis therapy [8]. Global evidence has demonstrated that continuous prophylaxis treatment led to better outcomes and Health-Related Quality of Life (HRQoL) than intermittent prophylaxis and on-demand treatment [3, 9–18]. Studies have indicated that prophylaxis therapy reduced the frequency of hemorrhages, maintains musculoskeletal functions, delays joint destruction, and reduced mortality [3, 9, 10, 12, 14, 16–18]. For instance, a study revealed that prophylaxis treatment led to a reduction of the annual number of total bleeds from 33.7 to 2.5 times and a decreasing of annual number of joint bleeds from 29.3 to 1.8 times by average among hemophilia children [3]. Long-term prophylaxis treatment at early ages could feasibly improve joint functions and prevent athropathy [17]. Other studies assessed the effect of prophylaxis therapy on HRQoL and found HRQoL score was significantly higher in the prophylaxis treatment group than in other treatment groups [9, 12, 19, 20].

In China, it was estimated more than 65,000 patients live with hemophilia [21]. Due to economic constraints, a great number of patients could not be able to obtain adequate prophylaxis treatment [22–24]. An international survey revealed that, among adult patients with haemophile in China, only 4.1% of them were treated by regular prophylaxis, and 21.6% of them were treated by on-demand plus short-term prophylaxis treatment, which was substantially lower than in other countries [23].

Although some local studies explored the association of prophylaxis treatment with patient-reported outcomes and HRQoL among adults with hemophilia in China [25–29], few studies focused on those connections among children patients [19, 30]. One study has found a smaller number of bleedings and higher joint health scores in the low-dose prophylaxis group than in the on-demand group among 34 children with severe HA [30]. Another study of 23 Chinese boys with HA found a 94% reduction in annual total bleeds and a significant improvement in quality of life with prophylaxis treatment than with on-demand treatment [19]. However, there is a paucity of evidence regarding the real-world distribution of treatment regimens and related comprehensive benefits on clinical outcomes and HRQoL among children with moderate and severe HA in China based on data from nationwide samples.

This study aimed to fill the gap in the literature using real-world data to compare the association of different treatment regimens with patient-reported outcomes and HRQoL of children with HA living in China.

## Methods

### Patients

In this study, patients were included by the following inclusion criteria: (1) Chinese patients who were diagnosed with HA; (2) patients with moderate HA (FVIII = 0.01 to ≤ 0.05 IU/mL) and severe HA (FVIII < 0.01 IU/mL);(3) patients without FVIII inhibitors; (4) patients no more than 18-years-old; and (5) patients and their caregivers provided informed consent.

To identify eligible patients across the country and conduct an investigation, we collaborated with the Hemophilia Home, which is the biggest hemophilia patient organization in China, having over ten thousand registered patients. Target patients were sampled from the registered patients of this organization.

### Data collection

HA children’s real-world data were collected from two parts and merged with each other. Firstly, patients’ demographic characteristics, patient-reported outcomes, and HRQoL were collected in July 2021 through a nationwide online cross-sectional survey embedded in the Hemophilia Home’s Apps. An invitation letter introducing the purpose and procedure of this survey was sent out to all registers of Hemophilia Home. The patients and their caregivers who agreed with the term and gave full informed consent were invited to fill out the questionnaire via Apps. Parents of children who are less than 8 years old or over 8 years old but unable to complete the questionnaire reported the related information for their children. Individual data on disease history, and patient-reported outcomes including bleedings, pains, target joints, and disability in the last 12 months were collected. EQ-5D-5 L scale was employed to measure the health-related quality of life among patients.

Secondly, patients’ use of FVIII was collected from the patient self-management database from the Hemophilia Home’s Apps for the same group of the participant in the national survey. Hemophilia Home encouraged registered patients to upload accurate, real-time information on the use of FVIII by scanning the QR code on the outer packing when infusing. After identifying the survey responders, their records of FVIII utilization from July 2020 to June 2021 were extracted from the patient self-management database.

**Table 1:** Characteristics of the patients with HA

Characteristic	All patients, n = 373	On-demand treatment, n = 82	short-term prophylaxis treatment, n = 273	long-term prophylaxis treatment, n = 18
**n (%)**	373(100.0)	82(22.0)	273(73.2)	18(4.8)
**Age groups, n (%)**				
Mean	7.3	7.2	7.3	7.3
0–4	136(36.5)	31(37.8)	102(37.4)	3(16.7)
5–9	116(31.1)	21(25.6)	84(30.8)	11(61.1)
10–14	90(24.1)	21(25.6)	66(24.2)	3(16.7)
15–18	31(8.3)	9(11.0)	21(7.7)	1(5.6)
**BMI, n (%)**				
Underweight	162(43.4)	40(48.8)	114(41.8)	8(44.4)
Normal	154(41.0)	30(36.6)	116(42.5)	7(38.9)
Overweight	58(15.6)	12(14.6)	43(15.8)	3(16.7)
**Social health insurance, n (%)**				
Yes	360(96.5)	78(95.1)	264(96.7)	18(100.0)
No	13(3.5)	4(4.9)	9(3.3)	0(0.0)
**Regional location, n (%)**				
East area	173(46.4)	36(43.9)	124(45.4)	13(72.2)
Mid area	95(25.5)	15(18.3)	77(28.2)	3(16.7)
West area	105(28.2)	31(37.8)	72(26.4)	2(11.1)
**Hemophilia severity, n (%)**				
Moderate	111(29.8)	31(37.8)	73(26.7)	7(38.9)
Severe	260(69.7)	50(61.0)	199(72.9)	11(61.1)
Missing	2(0.5)	1(1.2)	1(0.4)	0(0.0)
**Infectious blood disease, n (%)**				
No	352(94.4)	76(92.7)	258(94.5)	18(100.0)
Yes	18(4.8)	6(7.3)	12(4.4)	0(0.0)
Unknown	3(0.8)	0(0.0)	3(1.1)	0(0.0)

The Institutional Review Board Ethics Committee of Fudan University approved the study protocols (Ref no.: IRB#2022-02-0951).

### Treatment regimens

Treatment regimens were analyzed based on the records of FVIII utilization and patients’ body weights from the database. According to Chinese guidelines on the treatment of hemophilia (version 2020) published by the Chinese Society of Hematology Thrombus and Hemostasis Group and Chinese Hemophilia Cooperative Group [31], the standard dose of factor concentrates for long-term prophylaxis treatment was 10 IU/kg^-1^ of body weight and frequency was two times per week lasting for at least a year. After analyzing annual factor consumption and body weight for each patient, three treatment regimens were defined and classified by this standard from the guideline:

On-demand treatment: patients receive on-demand treatment only after the occurrence of bleeding.

Short-term prophylaxis treatment: patients receive prophylaxis treatment not up to the standard from the Chinese guideline. They might lack dose according to their body weights or not reach the required frequency (two times per week).

Long-term prophylaxis treatment: patients receiving prophylaxis treatment up to the standard. They must receive the standard dose according to their body weights and also takeinfusions two times per week.

**Table 2:** Comparison of patient-reported outcomes and HRQoL of patients with different regimens

Outcomes	All patients, n = 373	On-demand treatment, n = 82	short-term prophylaxis treatment, n = 276	long-term prophylaxis treatment, n = 18
**Annual bleeds, mean (SD)**	32.8(46.4)	63.0(65.6)	25.2(35.9)	9.8(16.3)
**Chronic pain, n (%)**				
Never	111(29.8)	13(15.9)	87(31.9)	11(61.1)
Occasionally	163(43.7)	33(40.9)	124(45.4)	6(33.3)
Sometimes	75(20.1)	22(26.8)	52(19.0)	1(5.6)
Frequently	23(6.2)	13(15.9)	10(3.7)	0(0.0)
Always	1(0.3)	1(1.2)	0(0.0)	0(0.0)
**Target joints, mean (SD)**	0.7(1.5)	0.7(1.4)	0.7(1.5)	0.3(0.6)
**Disability, n (%)**				
No	353(94.6)	75(91.5)	260(95.2)	18(100.0)
Yes	20(5.4)	7(8.5)	13(4.8)	0(0.0)
**EQ-5D, mean (SD)**	0.875(0.186)	0.804(0.255)	0.890(0.157)	0.952(0.125)
**EQ-VAS, mean (SD)**	78.06(23.82)	71.96(27.01)	79.25(22.64)	87.72(20.92)

### Outcome measures

#### Patient-reported outcomes

Consisting with the existing clinical definitions and standards in Chinese guidelines on the treatment of hemophilia (version 2020) [31], the related criteria were given to participants to define and report bleedings, target joints, and chronic pain.

Annual bleeds were defined as the total number of bleeds for each patient in a year. The results were derived from the patient’s self-reported bleeding times, which were collected in the survey using a single question “How many bleeds have you had in the past year?”

Chronic pain was defined as pain related to hemophilia from a persistent cause. It was asked with the question “During the past 12 months, have you experienced chronic pain due to hemophilia (like back pain, pain from sore joints, or arthropathy)?”. The answers were classified into 5 levels according to the frequency of occurrence: never (0% of the time), occasionally (less than 25% of the time), sometimes (25-50% of the time), frequently (50-75% of the time), and always (more than 75% of the time) and the ordinal variable was used for analysis.

Target joints were defined as the total number of swollen joints with limited movement and chronic synovitis. The question was “How many target joints have you had told by the doctors currently?”

**Table 3:** Multivariate regression results of treatment regimens effect on patient-reported outcomes and HRQoL.

Variables	Annual bleeds		Chronic pain		Target joints		Disability		EQ-5D		EQ-VAS	
	AME	95%CI	OR	95%CI	AME	95%CI	OR	95%CI	AME	95%CI	AME	95%CI
**Treatment regimens (vs. On-demand)**												
Short-term prophylaxis	-37.62***	-52.91 to -22.33	0.34***	0.21 to 0.55	-0.10	-0.21 to 0.40	0.58	0.23 to 1.64	0.08**	0.02 to 0.14	6.63*	0.10 to 13.26
Long-term prophylaxis	-52.27***	-79.26 to -34.28	0.08***	0.02 to 0.27	-0.27	-0.63 to 0.09	1.00	NA	0.12**	0.03 to 0.20	14.69*	2.32 to 27.06
**Age (vs. 0–4)**												
5–9	0.31	-10.50 to 11.11	3.63***	2.18 to 6.06	0.62***	0.35 to 0.89	2.79	0.26 to 29.27	0.03	-0.01 to 0.07	-0.57	-6.43 to 5.38
10–14	3.17	-10.34 to 16.67	3.09***	1.77 to 5.39	1.31***	0.91 to 1.71	10.64*	1.24 to 90.93	0.05*	0.01 to 0.09	0.64	-5.97 to 7.25
15–18	3.66	-10.66 to 17.97	16.20***	7.91 to 33.18	1.84***	1.27 to 2.41	92.16***	8.20 to 103.55	-0.07	-0.17 to 0.03	-9.61*	-18.91 to -0.30
**BMI (vs. Underweight)**												
Normal	3.24	-6.97 to 13.45	0.93	0.60 to 1.44	0.06	-0.20 to 0.32	0.53	0.13 to 2.02	0.01	-0.04 to 0.04	0.08	-5.23 to 5.40
Overweight	4.77	-7.72 to 17.27	1.30	0.67 to 2.50	0.69*	0.15 to 1.23	0.74	0.14 to 3.73	-0.04	-0.10 to 0.02	-5.03	-13.27 to 3.21
**Social health insurance (vs. Yes)**												
No	2.17	-19.55 to 23.89	0.66	0.34 to 1.30	0.04	-0.80 to 0.89	1.00	NA	0.01	-0.09 to 0.10	-1.38	-13.40 to 10.64
**Regional location (vs. East area)**												
Mid area	7.00	-4.15 to 18.14	2.38**	1.40 to 4.07	0.41*	0.05 to 0.76	1.28	0.35 to 4.62	-0.06**	-0.10 to -0.01	-3.10	-9.11 to 2.90
West area	-4.24	-15.00 to 6.53	2.96***	1.79 to 4.89	0.47**	0.12 to 0.82	1.16	0.32 to 4.09	-0.06*	-0.11 to -0.01	-3.04	-9.52 to 3.44
**Hemophilia severity (vs. Moderate)**												
Severe	2.63	-14.17 to 8.90	0.89	0.55 to 1.44	0.28	-0.03 to 0.59	2.75	0.82 to 9.22	0.01	-0.02 to 0.04	2.96	-2.27 to 8.20
**Infectious blood disease (vs. No)**												
Yes	-3.61	-21.11 to 77.84	0.51	0.15 to 1.71	-0.06	-0.55 to 0.44	1.00	NA	-0.02	-0.12 to 0.09	-0.59	-14.27 to 13.08

AME, average marginal effect; OR, odds ratio. 95%CI, 95% confidence interval

*p < 0 0.05. **p < 0 0.01. ***p < 0 0.001

Disability was defined as whether a patient was judged as statutory disabled. It was assessed by the question “Wereyou judged as the statutory disabled with a legal certificate or not?” and the dichotomous variable was generated. The legal certificate is accessible to make evaluations for patients and is broadly accepted as the standard of disability in China.

#### Health-related quality of life (HRQoL)

HRQoL of patients was measured by the EuroQol 5-Dimensions 5-Levels (EQ-5D-5L) index utility score and visual analogue scale (EQ-VAS). Firstly, a patient’s health is measured in five dimensions including mobility, self-care, usual activities, pain/discomfort, and anxiety/depression, and every dimension is divided into five levels, ranging from 1 to 5 as the score. Using the value set for the Chinese population [32], the scores of five dimensions could convert into the health utility score, which ranges from − 0.391 to 1. Secondly, EQ-VAS, a visual analogue scale, is used to assess the patient’ s health and reflect the worst health status to the best health status from 0 to 100.

**Table 4:** Multivariate regression results of treatment regimens effect for two subgroups of hemophilia severity

Variables	Annual bleeds (AME)		Chronic pain (OR)		Target joints (AME)		Disability (OR)		EQ-5D (AME)		EQ-VAS (AME)	
	Moderate	Severe	Moderate	Severe	Moderate	Severe	Moderate	Severe	Moderate	Severe	Moderate	Severe
**Treatment regimens (vs. On-demand)**												
Short-term prophylaxis	-32.78**	-38.68***	0.45*	0.29***	0.20	0.14	0.12	0.77	0.16**	0.05**	11.29**	5.56*
Long-term prophylaxis	-61.06***	-49.67***	0.03**	0.11***	0.11	-0.39	1.00	1.00	0.18***	0.07**	18.02**	12.18*
**Age (vs. 0–4)**												
5–9	-9.75	2.27	6.07***	3.60***	0.55*	0.62***	1.00	3.24	0.01	0.03	-11.02*	4.32
10–14	-10.99	8.55	1.49*	4.19***	1.00**	1.45***	1.00	9.58**	0.01	0.06	-6.4	2.18
15–18	-8.02	2.72	15.41**	22.60***	1.20**	2.03***	0.08	58.93***	-0.05	-0.09	-15.25*	-10.36*
**BMI (vs. Underweight)**												
Normal	9.60	1.25	1.17	0.84	0.02	0.06	0.23	0.64	-0.02	0.01	-2.75	2.06
Overweight	-14.32	11.30	2.16	1.13	0.15	0.88**	1.00	1.23	-0.03	-0.03	-10.24*	-2.52
**Social health insurance (vs. Yes)**												
No	1.87	5.45	0.28	0.82	0.28	-0.14	1.00	1.00	0.12	-0.02	6.94	-2.24
**Regional location (vs. East area)**												
Mid area	4.41	6.95	1.27	2.65***	0.01	0.50**	1.22	1.27	-0.09	-0.04*	-11.63*	0.97
West area	-18.24	0.71	3.16	2.90***	0.55	0.36*	1.00	1.57	-0.11	-0.03	-10.23*	-6.23
**Infectious blood disease (vs. No)**												
Yes	2.90	-3.49	0.02**	1.46	-0.66*	0.20	1.00	1.00	0.15	-0.08	22.59***	-10.57

AME, average marginal effect; OR, odds ratio. The results of 95% CI are omitted due to the limited room

*p < 0 0.05. **p < 0 0.01. ***p < 0 0.001

### Statistical analysis

Descriptive statistics were used to describe the patients’ characteristics, treatment regimens, patient-reported outcomes, and HRQoL. The mean and standard deviation for continuous variables and numbers and proportions for categorical variables are reported. To evaluate the differential association of treatment regimens with patient-reported outcomes or HRQoL among treatment regimens, the multivariate linear regression model was conducted for annual bleeds, target joints, EQ-5D-5 L utility scores, and EQ-VAS scores, and the logistic regression model for disability and the ordinal logistic regression for chronic pain were employed. Other related factors were controlled including age group, body mass index (BMI), health insurance, regional location, hemophilia severity, and having infectious blood disease.

The statistically significant level was 0.05. The Statistics Data Analysis (STATA) software, version 15.1 (StataCorp LLC, College Station, TX, USA) was used for all the analysis.

## Results

### Population characteristics

Real-world data of 373 children with HA without inhibitors from 29 provinces were collected. The characteristics of these patients are reported in Table 1. They were all boys with an average age of 7.3 years old. 15.6% of HA children were overweight. 69.7% of them were categorized as severe HA with factor activity less than 1% of the normal factor activity, and 29.8% as moderate with the factor activity between 1 and 5%. 18 patients were infected with blood diseases such as hepatitis. 96.5% of patients had social medical insurance and 46.4% lived in the Eastern region.

### Status quo of treatment regimens

According to the classification from the Chinese guideline and patients’ real-world data on FVIII use, only 4.8% of HA children received long-term prophylaxis treatment, 73.2% received short-term prophylaxis treatment, and 22.0% received on-demand treatment. HA children, who were not overweight, with severe hemophilia, without infectious blood disease, and lived in the Eastern regions were more likely to receive short-term or long-term prophylaxis treatment.

### Comparison of patient-reported outcomes and HRQoL among different regimens

Patient-reported outcomes and HRQoL of children with different regimens were shown in Table 2. Overall, for children with HA without inhibitors, the average annual bleeds were 32.8 times. 43.7% of them occasionally had chronic pain, and 29.8% of them never had chronic pain, whereas 6.5% of them frequently or always suffered from chronic pain. On average, every patient had 0.7 target joints and 5.4% of children were disabled. The mean health utility score among children with HA was 0.875 and the mean EQ-VAS score was 78.06.

The results of the multivariate regression models concerning the association of treatment regimens effect with patient-reported outcomes and HRQoL were presented in Table 3. Compared to on-demand treatment, prophylaxis treatment was a significant positive predictor of better patient-reported outcomes when holding other variables constant. Both patients with long-term or short-term prophylaxis treatment had significantly fewer annual bleeds than on-demand treatment (p < 0.001). The average annual bleeds of patients with long-term or short-term prophylaxis treatment were 9.8 and 25.2 times respectively. The results of the regression indicated that prophylaxis treatment significantly relieved patients’ chronic pain (p < 0.001). After any prophylaxis treatment, no children always suffered from chronic pain. Long-term prophylaxis treatment had a larger effect on reducing chronic pain than short-term prophylaxis treatment with 61.1% of patients never enduring chronic pain.

Children with prophylaxis treatment had fewer target joints and less probability of disability. However, neither of these differences among treatment regimens was statistically significant. The mean target joints for children with long-term prophylaxis treatment was 0.3, while children with other treatments had 0.7 target joints on average. No children with long-term prophylaxis treatment were disabled, while 8.5% of children with on-demand treatment and 4.8% of children with short-term prophylaxis treatment were disabled.

Treatment regimens were significantly and positively related to health utility scores and EQ-VAS scores. Patients receiving short-term and long-term prophylaxis treatment had higher scores of health utility and higher scores of EQ-VAS than patients receiving on-demand treatment. For patients with on-demand treatment, the mean health utility scores were 0.804, and EQ-VAS scores were 71.96. For patients with short-term prophylaxis treatment, the health utility scores (mean = 0.890, SD = 0.157) and EQ-VAS scores (mean = 79.25, SD = 22.64) were higher. For patients with long-term prophylaxis treatment, the mean health utility scores (mean = 0.952, SD = 0.125) and EQ-VAS scores (mean = 87.72, SD = 20.92) were highest than patients with the other two treatment regimens. Either difference in health utility scores and EQ-VAS scores among treatment groups was statistically significant (p < 0.01 and p < 0.05).

To show more comprehensive and detailed results, the multivariate regression models of treatment regimens effect for two subgroups of hemophilia severity were displayed in Table 3. Similar results were found compared to the results of the children with moderate and severe HA in Table 4. It was constant that the effect of prophylaxis treatment was significant representing fewer annual bleeds, less chronic pain, higher health utility scores, and higher EQ-VAS scores in both moderate and severe HA children. Also, prophylaxis treatment brought fewer target joints and less probability of disability to patients with prophylaxis treatment but the results were still not significant. The bigger effect of prophylaxis treatment on higher health utility scores and higher EQ-VAS scores in children with moderate HA than in children with severe HA.

## Discussion

This study described real-world treatment regimens of children with moderate and severe HA in China and comprehensively compared their patient-reported outcomes and health-related quality of life. It suggested that prophylaxis treatment was a significant positive predictor of better patient-reported outcomes and HRQoL. There were significantly fewer annual bleeds, lower frequency of chronic pain, and higher health utility scores and EQ-VAS scores for children with prophylaxis treatment than for children with on-demand treatment. Children with long-term prophylaxis treatment had the best health outcomes.

This study revealed that only 4.8% of children with HA were receiving full-dose prophylaxis treatment currently in China, which is far below that proportion in developed countries. Among children receiving any kind of prophylaxis treatment, over 90% haven’t reached the standard of the full dose of long-term prophylaxis treatment from the Chinese guideline. It was reported that nearly 94% of patients in the United Kingdom [33], 77% of patients in Canada, and 47% of patients in the United States [8] could receive full-dose prophylaxis treatment. The national variation in the use of prophylaxis treatment is considerable [34, 35] as well as the different regional distribution within China. It should be mentioned although prophylaxis is the standard of care and is generally used in children with HA, there might be an unmet need for utilization of the FVIII factors in real-world settings. With multiple participation, accessible health resources, favorable medical insurance benefit packages, and improved economic affordability are required in China [36].

Consistent with former studies, this study suggested that prophylaxis therapy decreases the frequency of hemorrhages, which is beneficial to prevent the progression of hemophilic complications [3, 9, 10, 14, 17]. Furthermore, we add new evidence that it also significantly relieves chronic pain related to hemophilia [37]. There was no statistical difference in the number of target joints and the probability of disability for children with different treatment regimens. Though some studies show that after prophylaxis therapy children suffered from less joint destruction or had higher scores on joint function scales [16, 17], while other studies did not find a statistically significant effect, which is our study find as well. Unlike adults, prophylaxis treatment has a limited and insignificant impact on reducing target joints, or promoting the range of joint motion for children [3, 9, 19]. The reason is that for children with different treatments, it takes a relatively long time to manifest the difference in the presence of target joints and even disability [3, 11, 13, 38].

Like other studies, our results also suggested that patients who receive prophylaxis treatment have a significantly higher quality of life [9, 12, 19, 20]. The mean health utility score of children with long-term prophylaxis treatment was 0.952, which was slightly higher than the results in other studies [12, 39] and nearly reached the same level as the average health utility score among healthy Chinese children [40]. It indicated that with regular infusions of FVIII as prophylactic therapy, children with HA could live a substantially normal life. At the same time, the bigger effect of prophylaxis treatment on higher quality of life in children with moderate HA than in children with severe HA may indicate that for patients with moderate hemophilia receiving prophylaxis treatment is more beneficial for their long-term outcomes.

This study has some strengths. As we know, it is the first study to comprehensively evaluate the influence of treatment regimens among children with moderate and severe HA in China by using four measures to assess the patient-reported outcomes and two measures to reflect the physical and psychosocial health status [9]. Real-world data of a relatively large sample were collected from two sources and merged. Follow-up records patient self-management database and cross-sectional survey of patient reports make it possible to precisely distinguish treatment regimens and estimate the impact on health outcomes. The limitation of our study should be mentioned. Sample bias may be present because we investigated children registered in Hemophilia Home who had better knowledge of HA and compliance. This may influence the estimation of the impact of prophylaxis treatment on improving patient-reported outcomes and quality of life.

## Conclusions

In conclusion, this study provides new evidence in China that children with HA, who received prophylaxis treatment have fewer annual bleeds, lower frequency of chronic pain, and higher health utility scores and EQ-VAS scores than on-demand treatment. Accessible long-term prophylaxis treatment with abundant infusion of FVIII should be evenly promoted as the optimal treatment regimen for children with moderate and severe HA in China and regular monitoring of its outcomes should be carried out in the future.

## Data Availability

The data that support the findings of this study are available from Hemophilia Home but restrictions apply to the availability of these data, which were used under license for the current study, and so are not publicly available. Data are however available from the authors upon reasonable request and with permission of Hemophilia Home.

## Abbreviations

HA: Hemophilia A
BMI: Body mass index
EQ‑5D‑5L: EuroQol 5-Dimensions 5-Levels
EQ-VAS: EuroQol 5-Dimensions 5-Levels visual analogue scale
HRQoL: Health‑related quality of life
